# Microtubule stabilization promotes the synthesis of type 2 collagen in nucleus pulposus cell by activating hippo-yap pathway

**DOI:** 10.3389/fphar.2023.1102318

**Published:** 2023-01-26

**Authors:** Xin Zhang, Shibin Shu, Zhenhua Feng, Yong Qiu, Hongda Bao, Zezhang Zhu

**Affiliations:** Division of Spine Surgery, Department of Orthopedic Surgery, Nanjing Drum Tower Hospital, The Affiliated Hospital of Nanjing University Medical School, Nanjing, China

**Keywords:** intervertebral disc degeneration, microtubule stabilization, mechanical stress, nucleus pulposus, intervertebral disc

## Abstract

Intervertebral disc degeneration (IDD) is the cardinal pathological mechanism that underlies low back pain. Mechanical stress of the intervertebral disc may result in a change in nucleus pulposus cells state, matrix degradation, and degeneration of the disc. Microtubules, which are components of the cytoskeleton, are involved in driving or regulating signal pathways, which sense and transmit mechano-transduction. Microtubule and the related proteins play an important role in the development of many diseases, while little is known about the role of microtubules in nucleus pulposus cells. Researchers have found that type II collagen (COL2) expression is promoted by microtubule stabilization in synovial mesenchymal stem cells. In this study, we demonstrated that microtubule stabilization promotes the expression of COL2 in nucleus pulposus cells. Stabilized microtubules stimulating Hippo signaling pathway, inhibiting YAP protein expression and activity. In addition, microtubules stabilization promotes the expression of COL2 and alleviates disc degeneration in rats. In summary, our study for the first time, identifies microtubule as a promising therapeutic target for IDD, up-regulating the synthesis of COL2 *via* Hippo-Yap pathway. Our findings may provide new insights into the etiologies and pathology for IDD, further, targeting of microtubule acetylation may be an effective strategy for the treatment of IDD.

## Introduction

Intervertebral disc (IVD) locates at between the vertebrae and is responsible for the load-bearing as well as stress-sharing in the spine. Intervertebral disc degeneration (IDD) is considered to be the principal cause of low back pain, which brings a heavy economic and social burden to the global healthcare system (Priyadarshani et al., 2016). The biomechanical loading involved in the pathogenesis of IDD have been elucidated through previous researches (Walter et al., 2011; Paul et al., 2012; Paul et al., 2013). Researchers have shown that a certain compressive force on the spinal motion segment can induce catabolism in the IVD, both *in vivo* and *in vivo* (Wang et al., 2007; Wuertz et al., 2009; Paul et al., 2013). In addition, overload-induced IVD degeneration could be attenuated by inhibiting NP cell apoptosis (Yamada et al., 2014). Therefore, exploring the internal relationship between mechanical force and IDD is beneficial to the biological targeted therapy of IDD.

Microtubules (MT), filamentous actin (F-actin) and intermediate filaments (IFs) together form a three-dimensional cytoskeleton, which mediates force transmission. Most microtubules are not static, they exhibit cycles of growth, shortening and regrowth. This dynamic instability allows MT responding to changes in internal or external conditions, and thus allows a cell to alter its cytoskeleton accordingly. Dynamic MT can generate pushing forces, which could maintain the cell shape under compressive forces (Brangwynne et al., 2006). However, how the MT involves in the NP cells and how the MT affect the ECM in disc still remains unknown. Microtubules, besides their role in cell shape maintenance, are involved in secretory transport mechanisms in collagen and proteoglycan synthesis (Benjamin et al., 1994). Previous study has shown that MT stability in synovial mesenchymal stem cells can promote cartilage formation and increase type Ⅱ collagen (COL2) expression (Li et al., 2021). Since IDD progression is triggered and accelerated by the degradation of ECM and the reduced level of COL2, we hypothesized that MT stabilization could also promote the synthesis of COL2 in the NP cells, decelerating IDD progression.

Yes-associated protein (YAP) and transcriptional coactivator with PDZ-binding motif (TAZ) serve an important role in biomechanical and mechanical signaling that affects cell proliferation and differentiation. YAP/TAZ read diverse biomechanical signals and transduce them into biological effects in a manner that is specific for each type of cell and mechanical-stress, which highlights the central role of YAP as universal mechano-transducers and mechano-effectors. Studies reveal that F-actin plays such an important role in the mechano-transduction between extracellular matrix and intervertebral disc cells (Hayes et al., 1999; Chen et al., 2004). Moreover, primary cilium, the key mechano-signaling sensor which mainly consists of MT, was critical for YAP activity. However, the mechanism that regulate YAP activity in the context of MT stabilization and destabilization in NP cells remains unanswered.

Hence, we investigated the role of MT stabilization in the synthesis of COL2 in NP cells, using docetaxel (MT stabilizer) and nocodazole (MT destabilizer). Our results suggest that MT stabilization of NP cells would be a potential therapeutic target for enhancing the synthesis of COL2 and alleviating IDD.

## Materials and methods

### NP cells isolation and culture

All the experimental protocols were approved by the Ethics Committee of Nanjing Drum Tower Hospital, the Affiliated Hospital of Nanjing University Medical School. With the informed consent of the patients, normal NP tissues were obtained from eight patients (Three males and five females, aged 12–23 years, mean age 17.8 years) who underwent surgery for congenital scoliosis. Degenerative NP tissues were collected from eight patients (Three males and five females, aged 40–65 years, mean age 53.7 years) who underwent posterior spinal fusion surgery. The human NP tissue samples were classified according to their degenerative grades (Song et al., 2018). After cutting into pieces, human NP tissues were enzymatically digested in 0.25% trypsin (Gibco; 15050065) and 0.2% type II collagenase (Gibco; 17101015) for 3 h. Following filtration and washing in PBS, the suspension was centrifuged. Isolated cells were grown in Dulbecco’s modified Eagle medium (DMEM) supplemented with 10% fetal bovine serum (FBS) (Gibco; 10099141) and 1% penicillin-streptomycin (Invitrogen; 10378016). The medium was replaced twice a week, and cells at 3–5 passage were used for the subsequent experiments.

### Western blot analysis

NP tissue and NP cells were lysed in the radioimmunoprecipitation assay (RIPA) buffer. The protein concentration was determined using a BCA kit (Thermo Scientific). 5% skim milk (Solarbio, D8340) was added to TBST (Solarbio, T1081) for 60 min to block membranes. After blocking, the membranes were incubated overnight at 4°C with the corresponding primary antibodies of Ace-tubulin (1:800; Cell Signaling Technology, 5335s), Collagen 2 (1:500; Proteintech, 28459-1-AP), SOX9 (1:1,000; Cell Signaling Technology, 82,630), Smad3 (1:1,000; Cell Signaling Technology, 9,523), YAP (1:1,000; Cell Signaling Technology, 14,074), p-YAP (1:1,000; Cell Signaling Technology, 13,008), TAZ (1:1,000; Cell Signaling Technology, 72,804), LATS1 (1:1,000; Cell Signaling Technology, 3,477), LATS2 (1:1,000; Cell Signaling Technology, 5,888), and GAPDH (1:1,000; Cell Signaling Technology, 5,174). Then, HP-linked secondary antibodies (1:5,000; Cell Signaling Technology, 7,074) were applied. The immunoblotting was detected by UVP ChemiDoc-It Imaging System (UVP, CA, USA) with the enhanced chemiluminescence detection kit (Thermo Fisher; 34,580) added to the membranes. Each blot was measured using ImageJ software for its integrated density.

### Quantitative polymerase chain reaction

A total RNA extract was prepared with Trizol (Invitrogen, 15,596,018). Following reverse transcription, the RNA was converted into cDNA using PrimeScript RT Master Mix Kit (TaKaRa, RR036A). Real-time polymerase chain reactions (RT-PCRs) were carried out using the TaKaRa SYBR Premix Ex Taq (RR420A) and the ABI StepOnePlus Real-Time PCR System (Applied Biosystems, CA, USA). By normalizing by GAPDH, fold changes of interested mRNAs were calculated. The primers were designed as follows:

Col2: 5′-AAG​GGA​CAC​CGA​GGT​TTC​ACT​GG-3′, 5′-GGG​CCT​GTT​TCT​CCT​GAG​CGT-3′; SOX9: 5′-AGC​GAC​AAC​TTT​ACC​AG-3′; 5′-GGA​AAA​CAG​AGA​ACG​AAA​C-3; GAPDH: ACA​GCA​ACA​GGG​TGG​TGG​AC-3′, 5′- TTT​GAG​GGT​ACA​GCG​AAC​TT-3′; YAP: 5′-GCT​AGA​TAA​AGA​AAG​CTT​T-3′, 5′-CCA​ATA​GTT​CCG​ATC​CCT​T-3′; TAZ: 5′- GAA​TCA​GCC​TCT​GAA​TCA​T-3′, 5′-GTC​TGA​AGA​TCT​GAT​CCC​T-3′; LATS1: 5′-CCACCCTAC CCAAAACATCTG-3′, 5′- CGC​TGC​TGA​TGA​GAT​TTG​AGT​AC-3′; LATS2: 5′-GCC​AAA​GAC​TTT​TCC​TGC​CA-3′, 5′- TCT​TTG​CTC​CCC​AGG​ACT​TT-3′.

### 5-Ethynyl-20-deoxyuridine assay

Using Ribobio’s EdU Reagent Kit (Guangzhou, China), the manufacturer’s instructions were followed to investigate cell proliferation. Following treatment with nocodazole and docetaxel for 3 days, the degenerative NPCs were exposed to 10 μM EdU for 2 h. Fluorescence images were captured using a fluorescence microscope (Zeiss, Heidelberg, Germany).

### Cell counting kit-8 assay

In accordance with the manufacturer’s instructions, the viability of NPCs was evaluated by using CCK8 assay ((Solarbio). We subsequently incubated each well for 2 h at 37°C with DMEM/F12 containing 10% (v/v) CCK-8 solution after washing with PBS. Microplate readers (Thermo Scientific, Logan, UT, United States) were used to measure absorbance at 450 nm.

### Immunofluorescence staining

Analyses of immunofluorescence were performed as previously described (Wu et al., 2017). We fixed cells and tissues attached to slides with 4% paraformaldehyde and permeabilized them with 0.5% Triton X-100 in PBS. After washing the slides in PBS and blocking with 2% bovine serum albumin (BSA) in PBS for 2 h at 37°C, the slides were incubated with primary antibodies against: Ace-tubulin (1:800), Col2 (1:500), or YAP (1:200). A second goat anti-rabbit antibody (Boster) was then applied at 37°C for 2 h after the slides. A DAPI staining solution (Beyotime, Nantong, China) was applied to the nuclei for 5 min before images were taken under an Olympus BX53 microscope (Zeiss, Heidelberg, Germany). Using ImageJ software (version 1.8.0), five randomly selected microscopic fields per slide were analyzed to determine the average gray value.

### Immunohistochemical staining

According to the manufacturer’s instructions, immunohistochemical staining and immunofluorescent analysis were performed. The serial sections were incubated overnight at 4°C with primary antibodies against Col2 (1:500; ab34712-Abcam) and Ace-tubulin (1:800; 5335s-Cell Signaling Technology). The slides were stained immunohistochemically with secondary antibodies conjugated with HRP, incubated at 37°C for 1 h. Photographs of sections were taken using a Zeiss fluorescence microscope (Zeiss, Heidelberg, Germany).

### Short interfering RNA transfection

The siRNA sequence was designed and synthesized to target the human YAP1 gene: 5-GGU​GAU​ACU​AUC​AAC​CAA​ATT-3 (Hippobio). We seeded NPCs in 6-well plates and grew them to a confluence of about 70%. Transfecting the cells with either 50 nM siRNA-YAP or a negative control, as directed by the manufacturer, took place for 12 h in Lipofectamine 3,000 (Thermo Fisher Scientific).

### Animal experiments

In accordance with the guidelines of the Institutional Animal Care and Use Committee at Nanjing University Medical School, all experiments were reviewed and approved by the committee. In the study, SD-Rats (10 weeks) were anesthetized with 10% chloral hydrate (0.3 mL/100 g) injected intraperitoneally. After placing the animals in a prone position, ethanol was applied to the tail skin for disinfection. A 21 G needle with a stopper was used to percutaneously puncture the discs Co6-7, Co7-8, and Co8-9 to the depth of 5 mm, after which the needle was rotated 360 degrees and held for 30 s. Four weeks after acupuncture, the caudal intervertebral discs of rats were examined by microCT and MRI. This study used MicroCT instead of X-ray to calculate the intervertebral disc height index (DHI), which is conducive to more intuitive indication of the changes in intervertebral disc height. The calculation method of DHI is as previously reported (Han et al., 2008). Degeneration grade of rat intervertebral disc was calculated and counted by MRI. We performed three IVDs on rats (Co6/7, Co7/8, and Co8/9) for the different treatments in order to evaluate the therapeutic effectiveness of microtubule stabilization for IDD. PBS, Nocodazole (3 μg/mL), and Docataxel (30 μg/mL) were administered intradiscally at the same total injection volume (2 μL) using 33-gauge needles (Hamilton, Benade, Switzerland) for all three IVDs (Co6/7, Co7/8, and Co8/9). During the 4 weeks treatment period, injections were given every week. After 4 weeks of treatment, microCT and MRI were performed again, and the intervertebral disc tissue of rats was stored in formalin for subsequent tissue staining.

### Histologic analysis

Using CO2 inhalation, all animals were sacrificed and the vertebral segments were dissected from the coccygeal segments 6–7, 7–8, and 8–9. Specimens were fixed in 10% formalin for 20 h at room temperature, then decalcified in an ethylenediaminetetraacetic acid (EDTA)-glycerol solution for 4 weeks. An embedding of decalcified tissue in paraffin, sectioning sagittally to a thickness of 5 μm. Hematoxylin-eosin was used to stain the slides of each disc, and a histological grading system was used to grade them (Han et al., 2008). Meanwhile, Masson staining and COL2 immunohistochemical staining was performed on the sectioned tissues.

### Statistical analysis

The means and standard deviations were calculated for all data. In GraphPad Prism 8, one-way ANOVA was used for the statistical analysis. In this study, statistical significance was defined as a *p* < 0.05.

## Results

### High expression of ace-tubulin and low expression of COL2 in human degenerated NP cells

**FIGURE 1:**
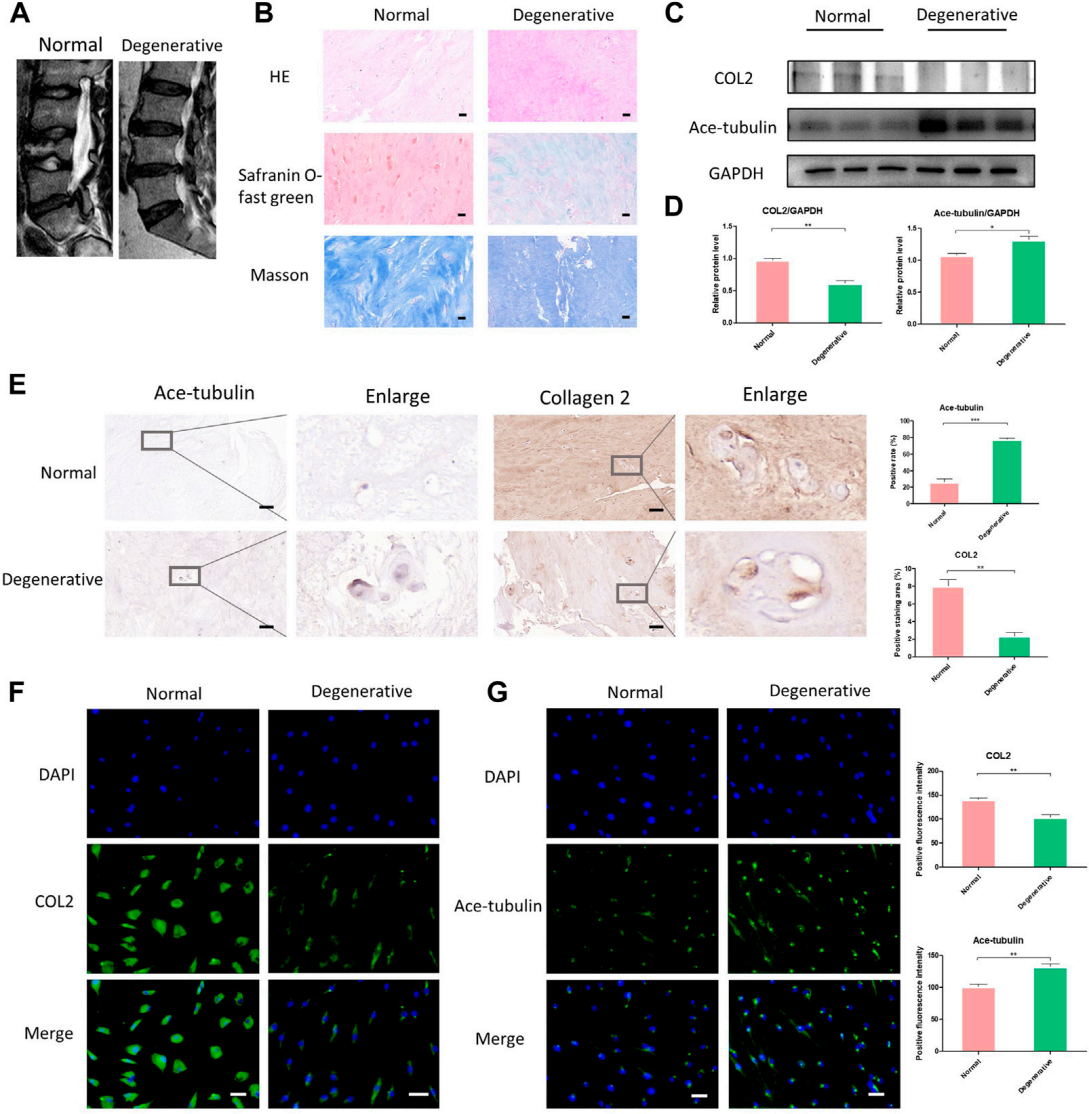
The differential expression of Ace-tubulin and type Ⅱ collagen (COL2) in human degenerated nucleus pulposus (NP) cells and normal NP cells. **(A)** T2 weighted imaging of human degenerative and normal intervertebral discs. **(B)** The HE staining, safranin O-fast green staining and Masson staining of human degenerated and normal nucleus pulposus tissues. **(C, E)** Western blot analysis and immunohistochemical staining of human degenerative and normal nucleus pulposus tissues. **(D)** Quantification of the data of **(C)**. *n* = 4. **(F, G)** Cell immunofluorescence staining of human degenerated and normal NP cells, Scale bar, 100 μm. Data are represented as the mean ± SEM. *p < 0.05, **p < 0.01, ***p < 0.001.

In order to harvest the degenerated NP cells, we collected human NP tissue samples from patients who underwent posterior transforaminal lumbar interbody fusion surgery due to lumbar spinal stenosis. The degenerated intervertebral discs all demonstrated low signal intensity (Pfirrmann grade Ⅳ or Ⅴ) in T2 Weight images of Magnetic Resonance (Figure 1A). The normal NP tissues in this study were derived from patients with congenital scoliosis who underwent posterior hemivertebra resection surgery (Figure 1A). The histological characteristics of degenerated NP tissues and normal NP tissues were evaluated using HE staining, Safranin O-fast green staining and Masson staining, to specify the different degeneration grade and collagen expression (Figure 1B). According to the HE staining, Safranin O-fast green staining and Masson staining, decreased number of NP cells and decreased expression of collagen were shown in the degenerated NP tissue. Since loss of COL2 presented a very important phenotype in the progression of IDD, we then compared the expression of COL2 in degenerated NP cells and normal NP cells. The lower expression of COL2 in degenerated NP cells was confirmed by Western blot, Cell immunofluorescence and Immunohistochemistry (Figures 1C, D, F).

Microtubules are involved in secretory transport mechanisms in collagen and proteoglycan synthesis. Thus we explored the stability of microtubules in degenerated NP cells. As previously reported, acetylated tubulin (Ace-tubulin) was used as a marker and quantitative index for microtubule stabilization since tubulin acetylation was found mainly on stable microtubules resistant to depolymerization (Westermann and Weber, 2003). Tubulin acetylation is a consequence of microtubule stability. Here, in the results of Western blot, cell immunofluorescence and immunohistochemistry, the expression of Ace-Tubulin was enhanced in degenerated NP cells (Figures 1C, E, F). For the first time, we observed increased Ace-tubulin in the NP cells, representing enhanced microtubule stability.

### MT stabilization promotes the expression of COL2 in NP cells

**FIGURE 2:**
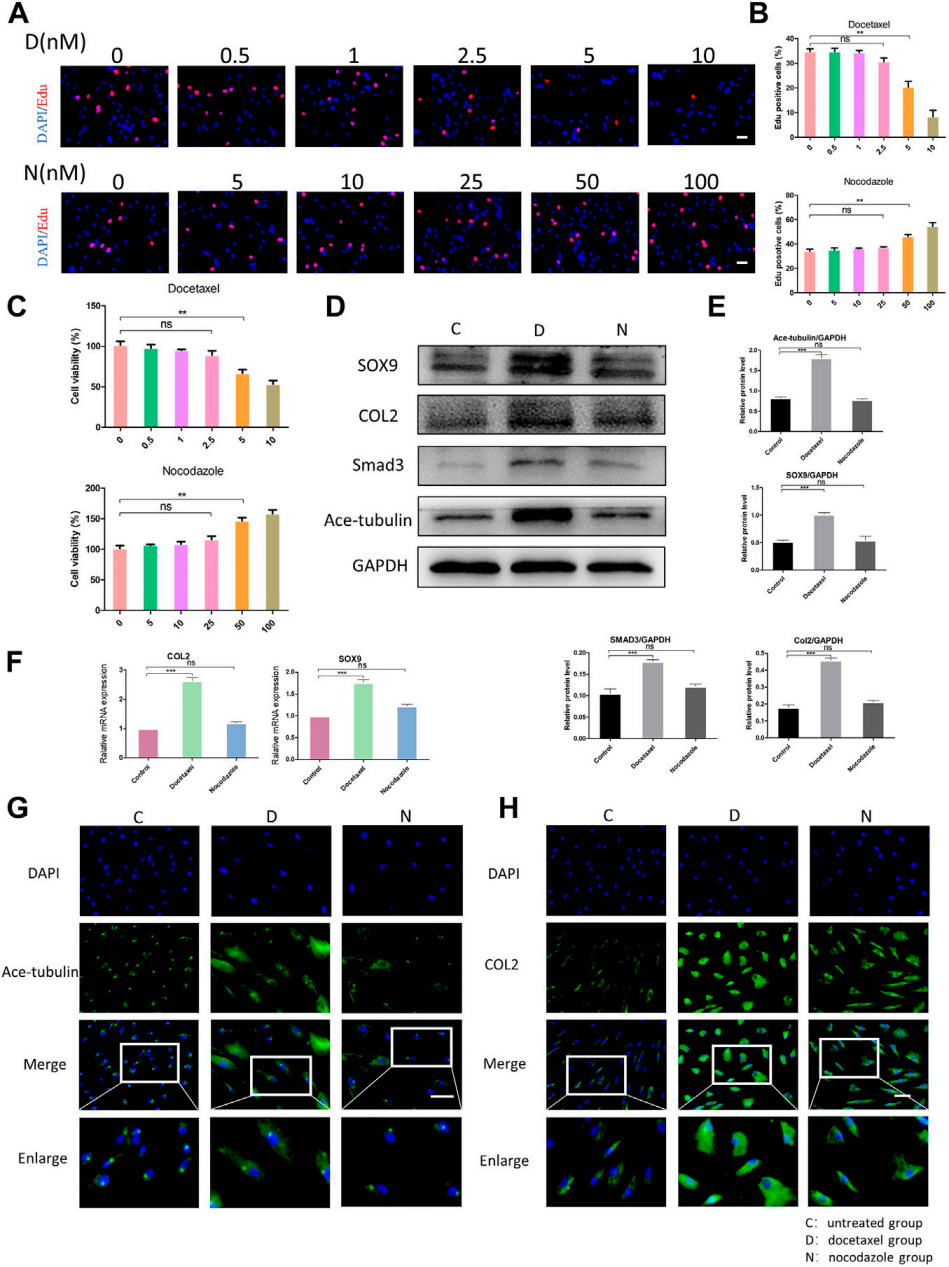
The effect of microtubule (MT) stabilization in the synthesis of COL2 in NP cells. **(A)** The EdU staining of degenerated NP cells after the treatment of different concentrations of docetaxel **(D)** (0.5, 1, 2.5, 5, 10 nM) and nocodazole (N) (5, 10, 25, 50, 100 nM) for 1 week. Scale bar, 100 µm. **(B)** Quantification of the data of **(A)**. *n* = 4. **(C)** Cell Counting Kit-8 Assay is used to detect cell viability. The cell viability of NP cells after the treatment of different concentrations of docetaxel (0.5, 1, 2.5, 5, 10 nM) and nocodazole (5, 10, 25, 50, 100 nM) for 1 week. *n* = 4. **(D)** Western blot analysis of Ace-Tubulin, COL2, SOX9, and SMAD3 expression in NP cells treated with docetaxel and nocodazole for 1 week. **(E)** Quantification of the data of **(D)**. *n* = 4. **(F)** Quantitative real time polymerase chain reaction (Q-PCR) of COL2 and SOX9 expression in NPCs treated with docetaxel and nocodazole for 1 week. **(G, H)** Cell immunofluorescence staining of Ace-Tubulin and COL2 expression in NP cells treated with docetaxel and nocodazole for 1 week. Scale bar, 100 µm. Data are represented as the mean ± SEM. *p < 0.05, **p < 0.01, ***p < 0.001.

Since the microtubule stability may regulate the cellular function of NP cells, we then investigated the effect of upregulation and the downregulation of microtubule stability on NP cells. Docetaxel, the microtubule stabilizer, and nocodazole, the microtubule depolymerizer, were used for the stabilization and destabilization of the microtubules, respectively. Regarding the cytotoxicity induced by high concentrations of docetaxel and nocodazole, we evaluated the proliferation and cell viability of NPCs treated with different concentrations of docetaxel and nocodazole using the CCK-8 and EdU staining. The EdU staining showed that the proliferation of NP cells was not significantly affected by docetaxel (0.5, 1, and 2.5 nM) or nocodazole (5, 10, and 25 nM), but was significantly inhibited by docetaxel at higher concentrations (5 and 10 nM) and enhanced by nocodazole at higher concentrations (50 and 100 nM) (Figures 2A, B). NP cells were assessed with the CCK-8 assay for cell viability (Figure 2C). Based on the results, 25 nM nocodazole slightly enhanced NP cells proliferation, while docetaxel within 2.5 nM had no significant effect on cell viability. The above results indicated that 2.5 nM docetaxel and 25 nM nocodazole were safe and efficacious for further investigation.

After treating NP cells with docetaxel and nocodazole for 1 week, the expression of Ace-tubulin was enhanced in NP cells treated with docetaxel compared to the untreated group, but no difference was found in NP cells treated with nocodazole, according to the results of Western blot and cell immunofluorescence (Figures 2D, E, G). As the MT stability increased using docetaxel, the results of Western blot, Q-PCR and cell immunofluorescence showed upregulated expression of COL2 (Figures 2D, F, H). Moreover, as the upstream regulators of COL2, the expression of SOX9 and Smad3 were also upregulated as microtubules stabilization increased (Figures 2D, E). As shown above, MT stabilization could promote the expression of COL2 in NP cells.

### MT stabilization inhibits the expression and activity of YAP/TAZ *via* hippo pathway

**FIGURE 3:**
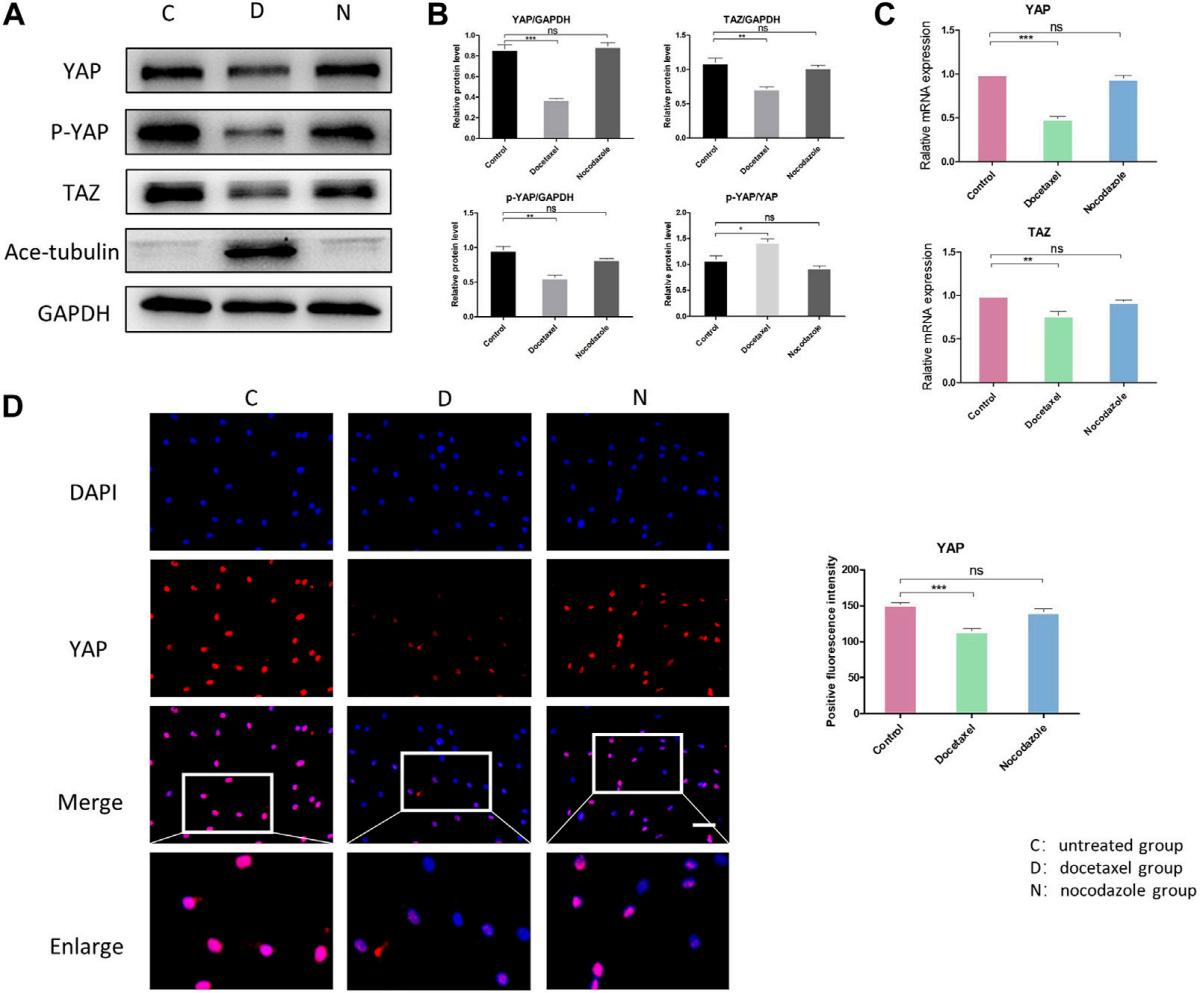
The effect of MT stabilization on YAP expression in NP cells. **(A)** Western blot analysis of YAP expression in NPCs treated with docetaxel and nocodazole for 1 week. **(B)** Quantification of the data of **(A)**. *n* = 4. **(C)** Q-PCR of YAP and TAZ expression in NP cells treated with docetaxel and nocodazole for 1 week. **(D)** Cell immunofluorescence staining of YAP expression in NP cells treated with docetaxel and nocodazole for 1 week. Scale bar, 100 µm. Data are represented as the mean ± SEM. *p < 0.05, **p < 0.01, ***p < 0.001.

YAP/TAZ plays a critical role in response to mechanical stress, perturbation of the cytoskeleton and matrix remodeling (Calvo et al., 2013). YAP/TAZ nuclear accumulation is a key determinant of their function by inducing gene expression through interactions with partner proteins; while YAP/TAZ could also be phosphorylated and inactivated, leading to their cytoplasmic retention and degradation. We investigated the expression and phosphorylation level of YAP/TAZ in NP cells to determine whether MT stabilization was involved in the expression and activity of YAP. The Western blot analysis showed that both total YAP and phosphorylated YAP were significantly decreased in NP cell after treatment of docetaxel, which was confirmed by Q-PCR (Figures 3A–C). Moreover, the proportion of phosphorylated YAP in total YAP ascended after treatment of docetaxel compared to the untreated group (Figures 3A, B), indicating that MT stabilization not only inhibits the expression of YAP, but promoting phosphorylation of YAP. MT destabilization by nocodazole treatment had no significant effect on total YAP and phosphorylated YAP in NP cells. The expression of TAZ decreased in docetaxel group and unchanged in nocodazole group, which was similar to that of YAP. The immunofluorescence results also confirmed that the docetaxel treatment significantly decreased total YAP, while no noteworthy change in YAP was observed after nocodazole treatment (Figure 3D). The above results showed that MT stabilization inhibited the expression and activity of YAP/TAZ in nucleus pulposus cells.

**FIGURE 4:**
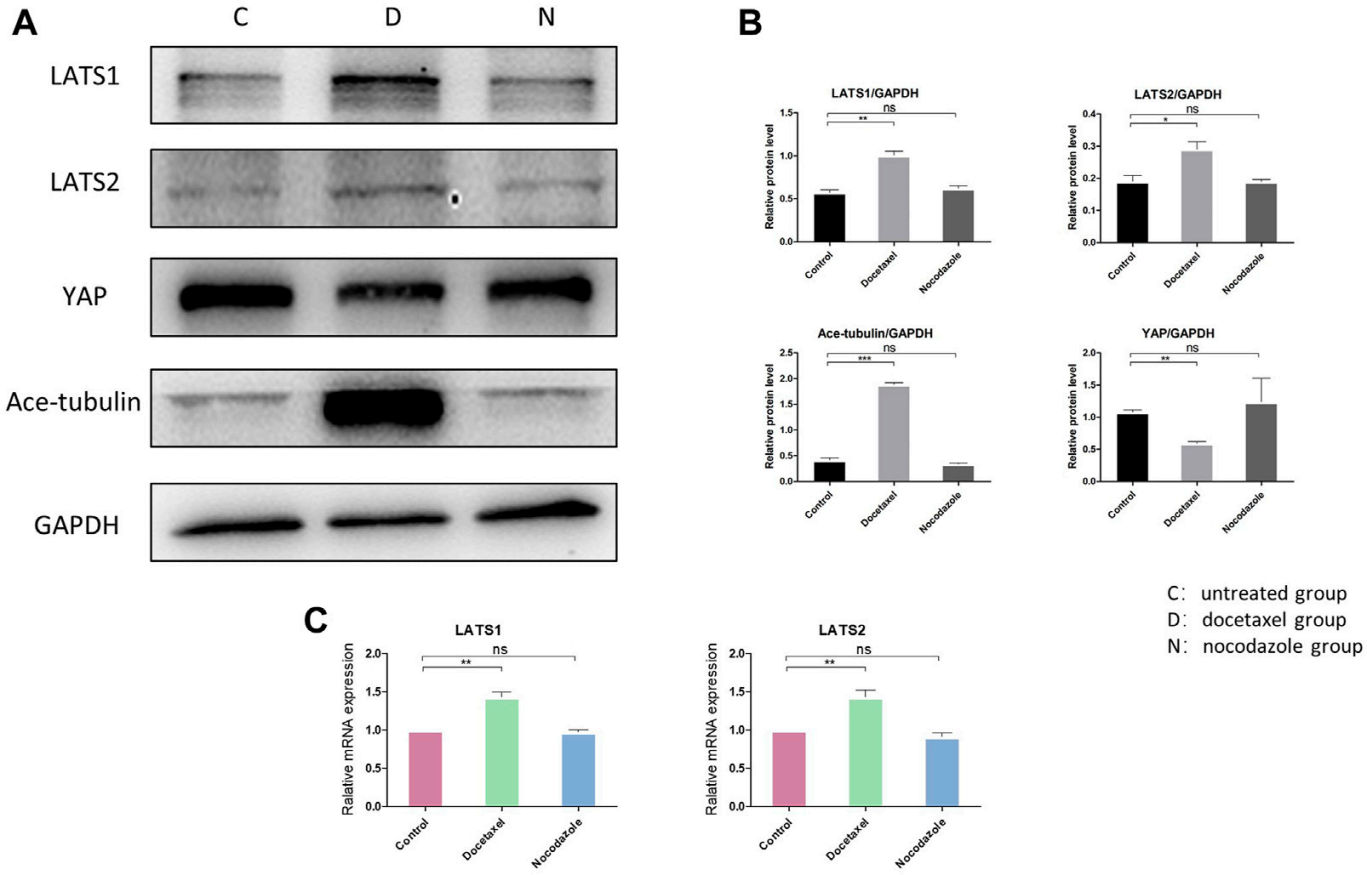
The effect of MT stabilization on Hippo pathway of NP cells. **(A)** Western blot analysis of LATS1, LATS2 and Ace-tubulin expression in NP cells treated with docetaxel and nocodazole for 1 week. **(B)** Quantification of the data of **(A)**. *n* = 4. **(C)** Q-PCR of LATS1 and LATS2 expression in NP cells treated with docetaxel and nocodazole for 1 week. Data are represented as the mean ± SEM. *p < 0.05, **p < 0.01, ***p < 0.001.

Hippo pathway regulates YAP/TAZ primarily through LATS kinases (LATS1 and LATS2)-mediated phosphorylation. Moreover, Hippo pathway is regulated by actin cytoskeletal factors, including capping proteins and cofilin (Gaspar and Tapon, 2014; Meng et al., 2016). A recent study also suggested that Patronin, a microtubule stabilizer, interacted with Hippo signaling in *Drosophila* (Yang and Choi, 2021). Therefore, we further investigated that whether the inhibition of YAP/TAZ by MT stabilization was mediated by Hippo pathway in NP cells. Our results in NP cells revealed that the docetaxel treatment significantly increased the expression of LATS1 and LATS2 (Figures 4A–C), which was consistent with previous findings that the components of the Hippo pathway promoted ciliogenesis and MT stabilization (Kim et al., 2014). In addition, the expression of LATS1/2 was not affected when NP cells were treated with nocodazole. Meanwhile, the enhanced expression of Ace-tubulin and inhibited expression of YAP was also found after docetaxel treatment by Western blot analysis. The above results illustrated that MT stabilization inhibited the expression and activity of YAP/TAZ *via* regulating Hippo pathway.

### YAP mediates MT stabilization to promote the synthesis of COL2

**FIGURE 5:**
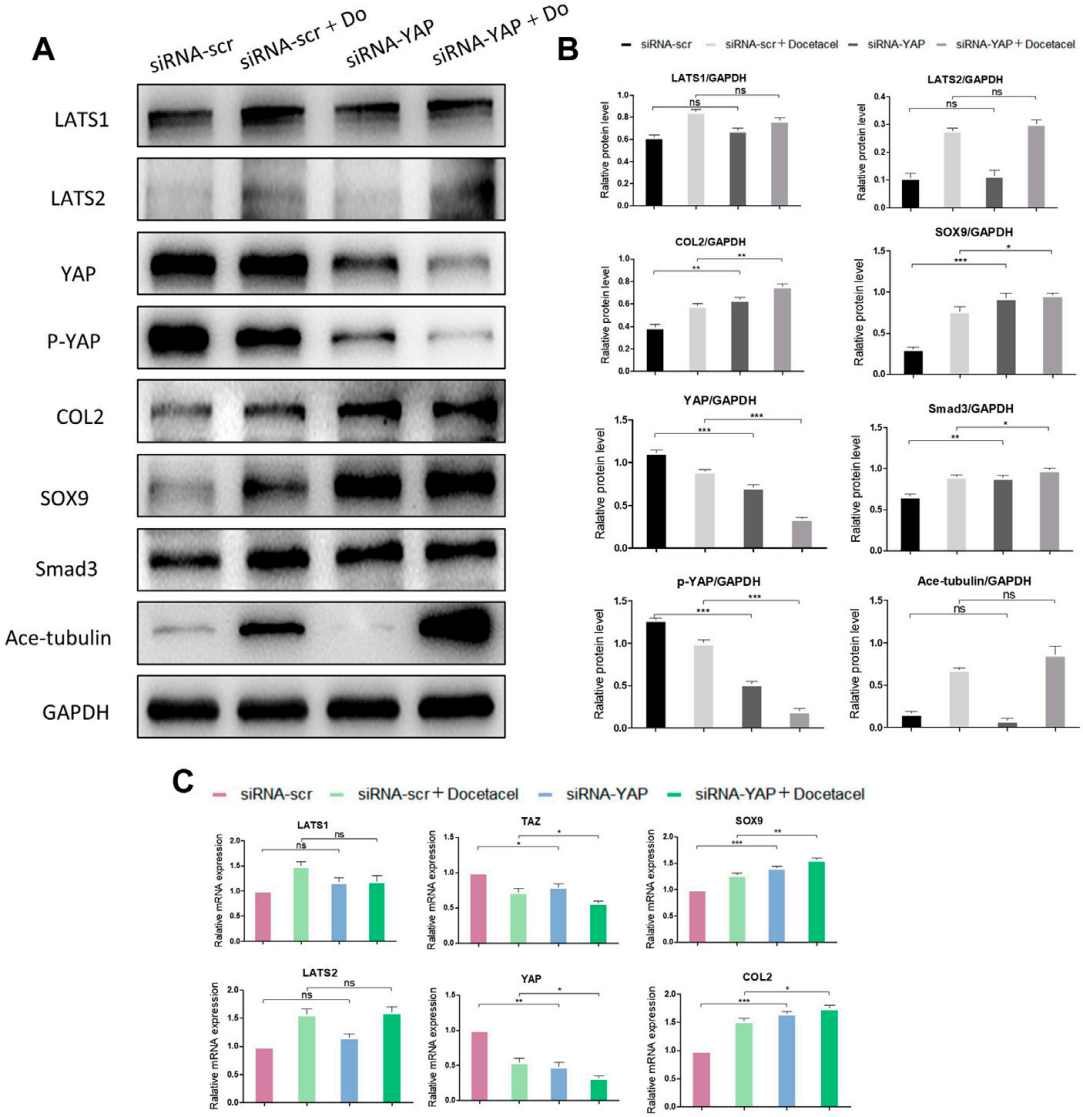
Knockdown of YAP promotes the synthesis of COL2 in NP cells. **(A)** Western blot analysis of Ace-Tubulin, phosphorylated YAP, YAP, LATS1, LATS2, COL2, Smad3 and SOX9 in NP cells transfected with siRNA-YAP and treated with docetaxel for 1 week. **(B)** Quantification of the data of **(A)**. *n* = 4. **(C)** Q-PCR of YAP, TAZ, LATS1, LATS2, COL2, and SOX9 in NP cells transfected with siRNA-YAP and treated with docetaxel for 1 week. Data are represented as the mean ± SEM. *p < 0.05, **p < 0.01, ***p < 0.001.

**FIGURE 6:**
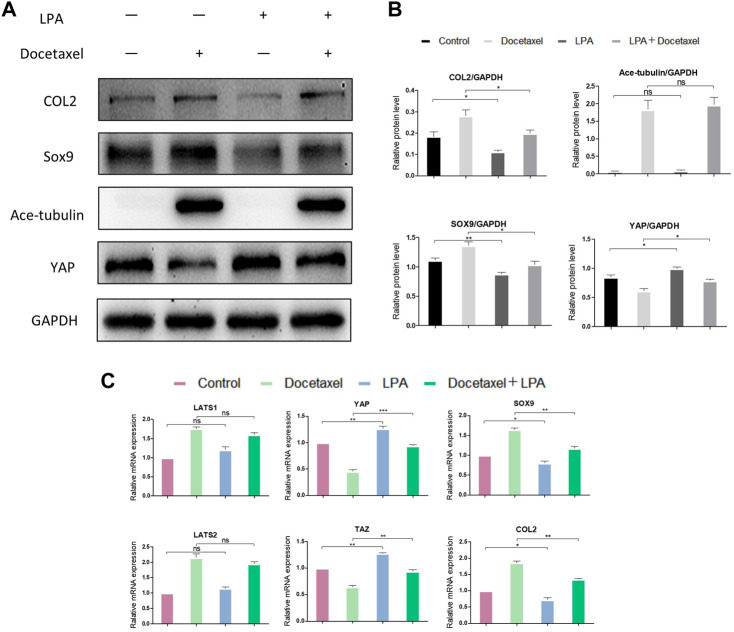
The effect of YAP activation on the synthesis of COL2 in NP cells. **(A)** Western blot analysis of Ace-Tubulin, YAP, COL2 and SOX9 in NP cells treated with lysophosphatidic acid (LPA) and treated with docetaxel for 1 week. **(B)** Quantification of the data of **(A)**. *n* = 4. **(C)** Q-PCR of LATS1, LATS2, TAZ, YAP, COL2 and SOX9 in NP cells treated with LPA and treated with docetaxel for 1 week. Data are represented as the mean ± SEM. *p < 0.05, **p < 0.01, ***p < 0.001.

To clarify whether YAP plays a role in the up-regulated COL2 expression promoted by MT stabilization, YAP was knocked down by small interfering RNA (siRNA). As the total and phosphorylated YAP both decreased following YAP siRNA transfection, the nucleus pulposus related proteins, including COL2, SOX9 and Smad3, were all upregulated with and without docetaxel treatment; while the expression of LATS1, LATS2 and Ace-tubulin were not significantly altered (Figures 5A, B). Moreover, Lysophosphatidic acid (LPA) was utilized to promote the nucleus re-localization of YAP in the NP cells, which would up-regulate the activity of YAP (Yu et al., 2012). The results revealed that the increased expression of COL2, SOX9 and Smad3 by docetaxel were diminished with concurrent treatment of LPA, while the LPA treatment alone to the NP cells also significantly decreased the expression of COL2, SOX9 and Smad3 (Figures 6A–C). Therefore, the synthesis effect of COL2 induced by MT stabilization was *via* the regulation on YAP.

### MT stabilization alleviate IDD *in vivo*

**FIGURE 7:**
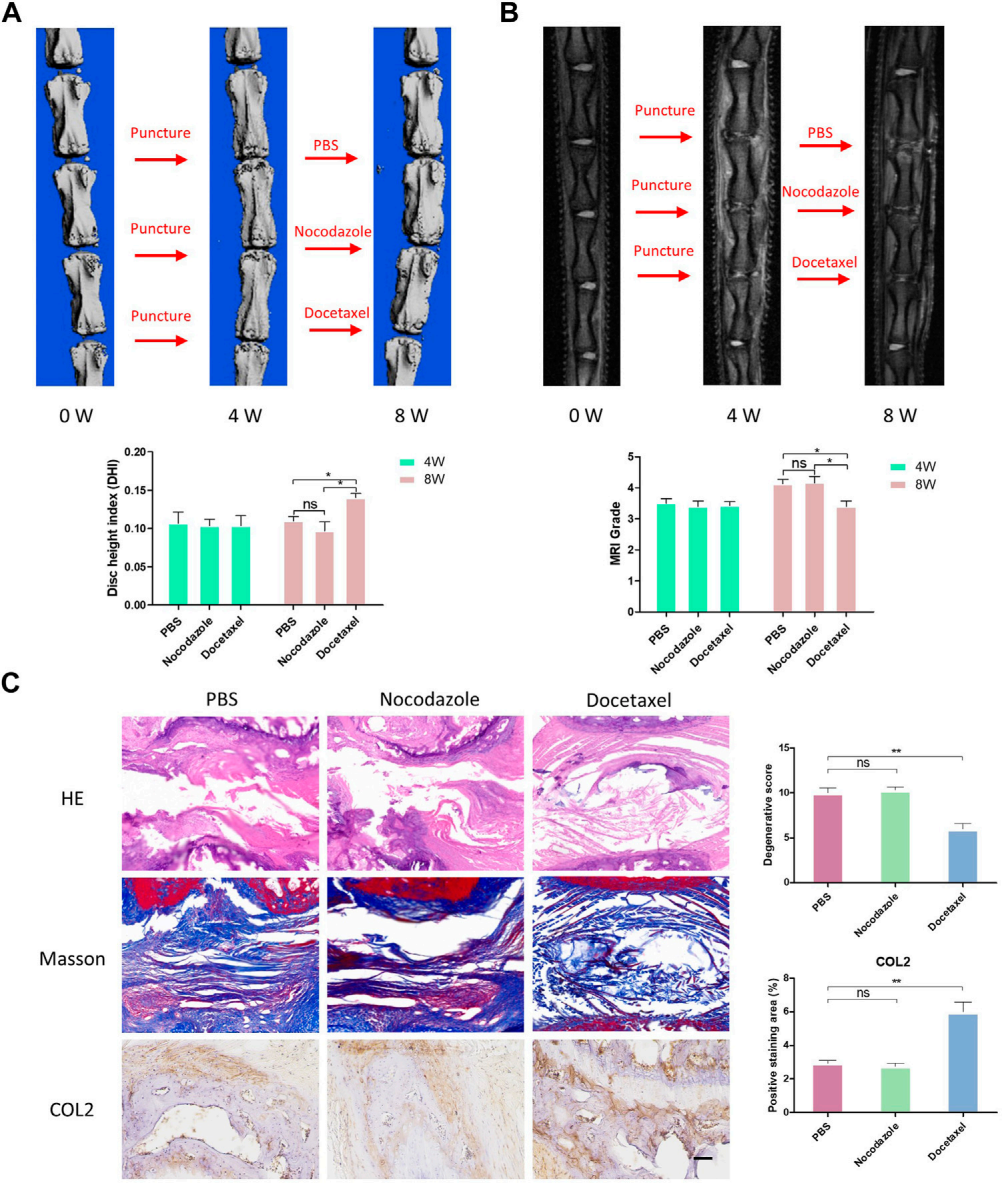
MT stabilization alleviates intervertebral disc degeneration (IDD) by promoting the synthesis of COL2 *in vivo*. **(A)** After 4 weeks of acupuncture and 4 weeks of treatment with PBS, nocodazole, and docetaxel, 3D micro-CT reconstruction and disc height of rat caudal IVD were collected and measured. **(B)** After 4 weeks of acupuncture and 4 weeks of treatment with PBS, Nokotazol and docetaxel, MRI and disc degenerative degree (low signal ratio in disc) of rat caudal IVD were collected and measured. **(C)** HE staining, Masson staining and COL2 immunohistochemistry staining of rat caudal IVD after treatment with PBS, Nocodazole and Docetaxel for 4 weeks. Data are represented as the mean ± SEM. *p < 0.05, **p < 0.01, ***p < 0.001.

To further investigate the therapeutic effects of MT stabilization on IDD, we used a classic rat caudal intervertebral disc acupuncture animal model for this study. PBS, nocodazole and docetaxel were injected into three consecutive independent intervertebral discs of each rat 4 weeks after acupuncture. MRI and microCT examinations of rat IVD were performed at 4 weeks after the modeling and 4 weeks after the treatment to determine the degree of degeneration. A percent disc height index (%DHI) was calculated based on the microCT results. With a low percent DHI in all rats 4 weeks after needle puncture, the intervertebral disc showed intervertebral space stenosis or collapse, indicating degenerative changes of IVD (Figures 7A, B). After 4 weeks of treatment with docetaxel and nocodazole, DHI in docetaxel group recovered significantly, while DHI in control group and nocodazole group did not increase significantly (Figure 7A). In addition, the Pfirrmann score was derived from MRI results and used to assess the degree of IDD. Four weeks after needle puncture, the Pfirrmann scores of IVD in all rats showed no significant difference. After 4 weeks of treatment with docetaxel and nocazole, Pfirrmann scores were significantly lower in the docetaxel group compared to the PBS group and the nocodazole group. Intervertebral discs were collected for HE staining, Masson staining, and COL2 immunohistochemical staining after treating rats for 4 weeks. The results showed that the Docetaxel group had a significantly better degenerative score than the PBS group and the Nocodazole group, and the expression of COL2 was also significantly increased compared with PBS group and Nocodazole group (Figure 7C).

## Discussion

**FIGURE 8:**
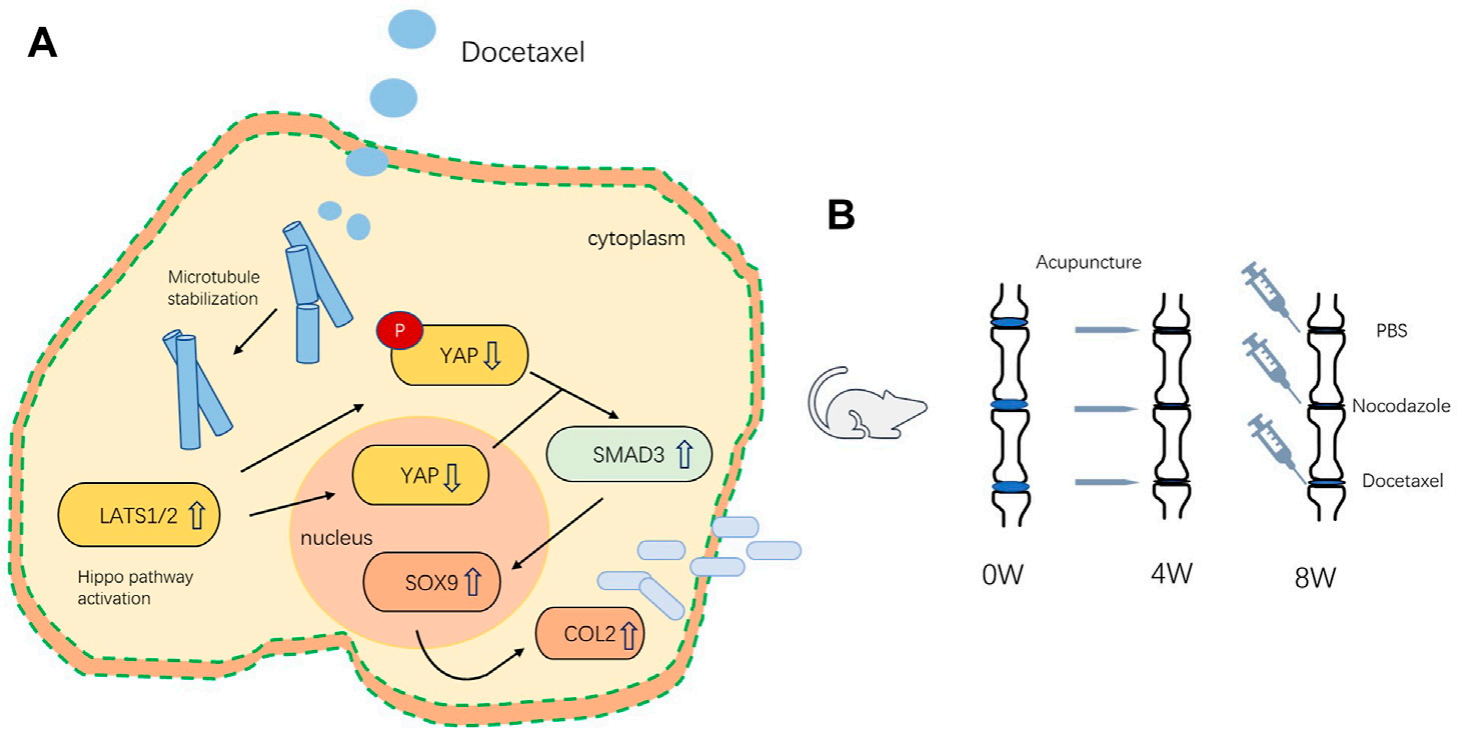
**(A)** Schematic of the mechanism by which microtubule stabilization promotes the synthesis of COL2 in NP Cell. **(B)** Schematic illustration of rat intervertebral disc modeling and treatment.

Microtubules outline the overall shape of cells by serving as the supports for morphologies such as axons and cilia (Goodson and Jonasson, 2018). Microtubules could also feel tension at the cell membrane, where they are sometimes coupled to motor proteins (Ligon et al., 2001), but the role of microtubule on mechanical force related degenerative diseases still remains unclear. In this study, we demonstrated that MT stabilization promoted the expression of Col2 and Sox9 in nucleus pulposus cells by stimulating Hippo pathway and inhibiting YAP expression and activity. MT stabilization promoted the expression of Col2 and alleviates disc degeneration *in vivo* in rats (Figure 8).

MT stabilization has been reported to have a significant effect on promoting the chondrogenic differentiation of Synovial mesenchymal stem cells derived from human osteoarthritis synovium (Li et al., 2021). However, few study investigated the relationship between microtubule and IDD. For the first time, we found that microtubule stabilization could promote the expression of chondrogenic makers, Col2 and Sox9, suggesting that MT stabilization would be a promising strategy for alleviating IDD. The length of primary cilia were found to decrease significantly both in annulus fibrosus cells and NP cells during IDD (Li et al., 2020), suggesting the potential impact of microtubule on IDD. Primary cilia are highly conserved microtubule-based organelles that project from the cell surface into the extracellular environment. Studies from bone and cartilage have demonstrated that primary cilia play critical roles during cell mechano-sensation and mechano-transduction (Goetz and Anderson, 2010; Ruhlen and Marberry, 2014). Li et al. (2020) further proved that cilia loss not only caused cell apoptosis in NP cells, but also lead to decreased Col1α1 and Col2α1 levels and increased MMP3 levels, suggesting that primary cilia are critical for the regulation of disc degeneration. Since primary cilia are microtubule-based organelles, the previous studies supported our results that MT stabilization would being positive benefit to the intervertebral disc.

Similar to the degeneration of intervertebral disc, MT destabilization has been reported to be one of the possible pathology of neurodegeneration, leading to Parkinson’s Disease (Cartelli and Cappelletti, 2017); on the other hand, MT stabilization can block degeneration of axon and even enable axon regrowth, making the MT-based therapy a promising option for Parkinson’s Disease (Baas and Ahmad, 2013). The use of MT stabilizer, Paclitaxel, has showed a beneficial effect by rescuing the PARK2 mutation-derived alterations of MT assembly in human neurons (Ren et al., 2009). In this study, we chose docetaxel as the MT stabilizer, which shared a similar mechanism with paclitaxel: the promotion of microtubule assembly and inhibition of microtubule disassembly. Our rat IDD model also proved the ability of MT stabilizer in blocking the disc degeneration and in promoting the expression of Col2. However, the benefits do not come without potential risks; since MTs need to be remodeled during the entire lifespan, MT stabilizers would block the dynamic behavior of MT and therefore affect MT-dependent functions in many aspects. Moreover, considering the difficulty of docetaxel delivery to intervertebral disc, the use of MT-interacting peptides (Fleming et al., 2011), which would modulate MT assembly while having better chances not to cause side effects, could be a helpful tool for physicians and researchers.

Acetylated microtubules have been considered to be stable, long-lived microtubules (Szyk et al., 2014). The traditionally prevailing model is that tubulin acetylation is a consequence of microtubule stabilization, however, in the living cells, the mechanical force could make acetylated microtubules more long-lived. At the same time, longer-lived microtubules are still more likely to experience mechanical stress, thus further accumulating acetylation marks (Janke and Montagnac, 2017). It’s very interesting that the MT depolymerizer, nocodazole, showed no significant effect on the expression of Ace-tubulin, YAP/TAZ and Col2 according to our data. Nocodazole is a small molecule that binds soluble tubulin and prevents its polymerization, working as a microtubule-destabilizing agent (Gudimchuk and McIntosh, 2021). Recently, Xu et al. (2017) found that after treating cells with nocodazole, dynamic microtubules were depolymerized, and most remaining microtubules displayed the typical characteristics of long-lived microtubules with high levels of acetylation, they named those long-lived microtubules as nocodazole-resistant microtubules. The data from Xu et al. (2017)’s study proved that nocodazole could depolymerize dynamic microtubules but the remining long-lived microtubules were still highly bent, acetylated, and detyrosinated and microtubule number was similar in control cells compared to the nocodazole treated cells. Xu’s study supported our finding that nocodazole does not necessarily decrease the expression of Ace-tubulin in degenerated NP cells. It could possibly attribute to the long-lived nature of microtubules in degenerated NP cells since the degenerated NP cells suffers more mechanical force. Similarly, since the long-lived microtubules was difficult to be depolymerized by nocodazole, the nocodazole treatment may not change the YAP/TAZ expression. Our data is supported by previous report that disruption of microtubules with nocodazole did not alter YAP/TAZ localization (Dupont et al., 2011).

YAP activity and subcellular localization are influenced by changes in the cytoskeleton and in cell shape (Gao et al., 2017). In NP cells, specifically, Zhang et al. (2021) demonstrated Hippo pathway was suppressed in the early stages of disc injury, and that YAP and F-actin activity decreased gradually with age in natural IDD. Our results revealed that the stabilization of MT, another component of cytoskeleton, would lead to the decreased expression of YAP and the increased proportion of YAP phosphorylation, which represented the decreased activity of YAP. YAP levels in NP tissue has been reported decreased gradually with time, suggesting that they are closely related to the development of IDD (Ding et al., 2022). In addition, consistent with our findings, Li et al. reported that MT stabilization inhibited the expression of YAP in Synovial mesenchymal stem cells to drive chondrogenesis. The precise mechanisms that how MT stabilization could suppress the activity of YAP has not been illustrated. Actin stress fibres are reported to be required for YAP/TAZ activity (Dupont et al., 2011), while Enomoto (1996) confirmed that taxol, a MT stabilization drug, could suppress the formation of actin stress fibres. Moreover, YAP activity is inhibited upon mechanical stress and microtubules are stabilized under tension (Dupont et al., 2011; Hamant et al., 2019). Taken together, MT stabilization may inhibit YAP/TAZ activity by interactions between cytoskeleton components.

The Hippo pathway controls the nuclear localization and stability of YAP/TAZ, playing important roles in maintaining homeostasis and tissue regeneration. Studies have shown that the activity of cytoskeleton are involved in the regulation of Hippo signaling pathways (Zhang et al., 2021). In our study, YAP de-activation was accompanied by upregulation of LATS1/2, indicating that Hippo signaling pathway acted as the upstream negative regulator of YAP/TAZ in NP cells when microtubule stabilization was promoted. Researches have provided a variety of mechanisms that regulating YAP/TAZ, including classic Hippo signaling pathway, ROCK signaling, TGF-β signaling, Snail/Slug binding interactions and Hedgehog signaling pathway. In the classic Hippo pathway, LATS1/2 phosphorylate the YAP/TAZ, which results in their cytoplasmic retention and degradation in the proteasome. In contrast, Zhang et al. reported that YAP activation was accompanied by upregulation of LATS1 in young rats intervertebral discs, they explained this finding as the YAP-LAST negative feedback loop which could limit the hyperactivity of YAP, but their conclusion may need further validation (Zhang et al., 2018).

In this study, the activity of YAP could regulate the synthesis of Col2, which serves as a marker of disc regeneration. For the first time, we observed that the YAP agonist (LPA) could suppress the synthesis of Col2 while YAP knockdown promoted synthesis in NP cells. Increased YAP expression levels has been reported to increase osteogenesis and decrease adipogenesis of mesenchymal stem cells and initiate dedifferentiation of chondrocytes (Zhong et al., 2013), and thus the decreased YAP activity may indicate chondrogenesis. Moreover, Zhong et al. treated mesenchymal stem cells and chondrocytes with Cytochalasin D, they found that Cytochalasin D could repressed nuclear YAP accumulation and thus result in MSC adipogenesis and the maintenance of the chondrocyte phenotype. The regulation of YAP on Col2 may rely on Smad3. Wei et al. inhibited Smad in degenerated NP cells and found the Smad inhibitor could abolish the effect of YAP. Our data also demonstrated increased Smad3 expression in conditions of both MT stabilization and YAP knockdown, and Smad3 has been reported as a key regulator of Col2 (Chen et al., 2012). In addition, in our *in vivo* study, the recovery of disc height, the improvement of MRI signal and the up-regulated Col2 expression after the injection of docetaxel into the caudal disc of rats demonstrated the therapeutic potential that targeting microtubule.

It is very interesting that we observed increased ace-tubulin in degenerated NP tissues derived from patients with IDD, which has not been reported in previous studies. The acetylation of tubulin is a consequence of microtubule stabilization since artificially chemical stabilization of microtubules leads to acetylation of tubulin (Palazzo et al., 2003). The mechanical force exposed to the cells could lead to depolymerization of microtubule, while acetylation-induced increase in flexibility would allow microtubules to better resist mechanical force, serving as a self-repair mechanism and making the acetylated microtubules more long-lived (Xu et al., 2017). Moreover, longer-lived microtubules are more likely to experience mechanical stress, further accumulating Ace-tubulin (Schaedel et al., 2015). Thus, the increased Ace-tubulin in degenerated NP tissues is probably due to the mechanical stress on intervertebral discs, and the MT stabilization may participate in a “self-regeneration” mechanisms during IDD since MT stabilization could promote synthesis of Col2.

In summary, our study for the first time, identifies microtubule stabilization as a promising therapeutic target for IDD, up-regulating the synthesis of Col2. Moreover, Hippo-Yap pathway participates in the regulation of Col2 in the context of stabilized microtubule. Our findings may provide new insights into the etiologies and pathology for IDD, further, targeting of microtubule acetylation may be an effective strategy for the treatment of IDD.

## Data Availability

The raw data supporting the conclusion of this article will be made available by the authors, without undue reservation.
