# Supplementary figures and images for: Microtubule stabilization promotes the synthesis of type 2 collagen in nucleus pulposus cell by activating hippo-yap pathway
Source: Front Pharmacol. 2023 Jan 26;14:1102318. doi: 10.3389/fphar.2023.1102318 (PMC9909034; doi:10.3389/fphar.2023.1102318)

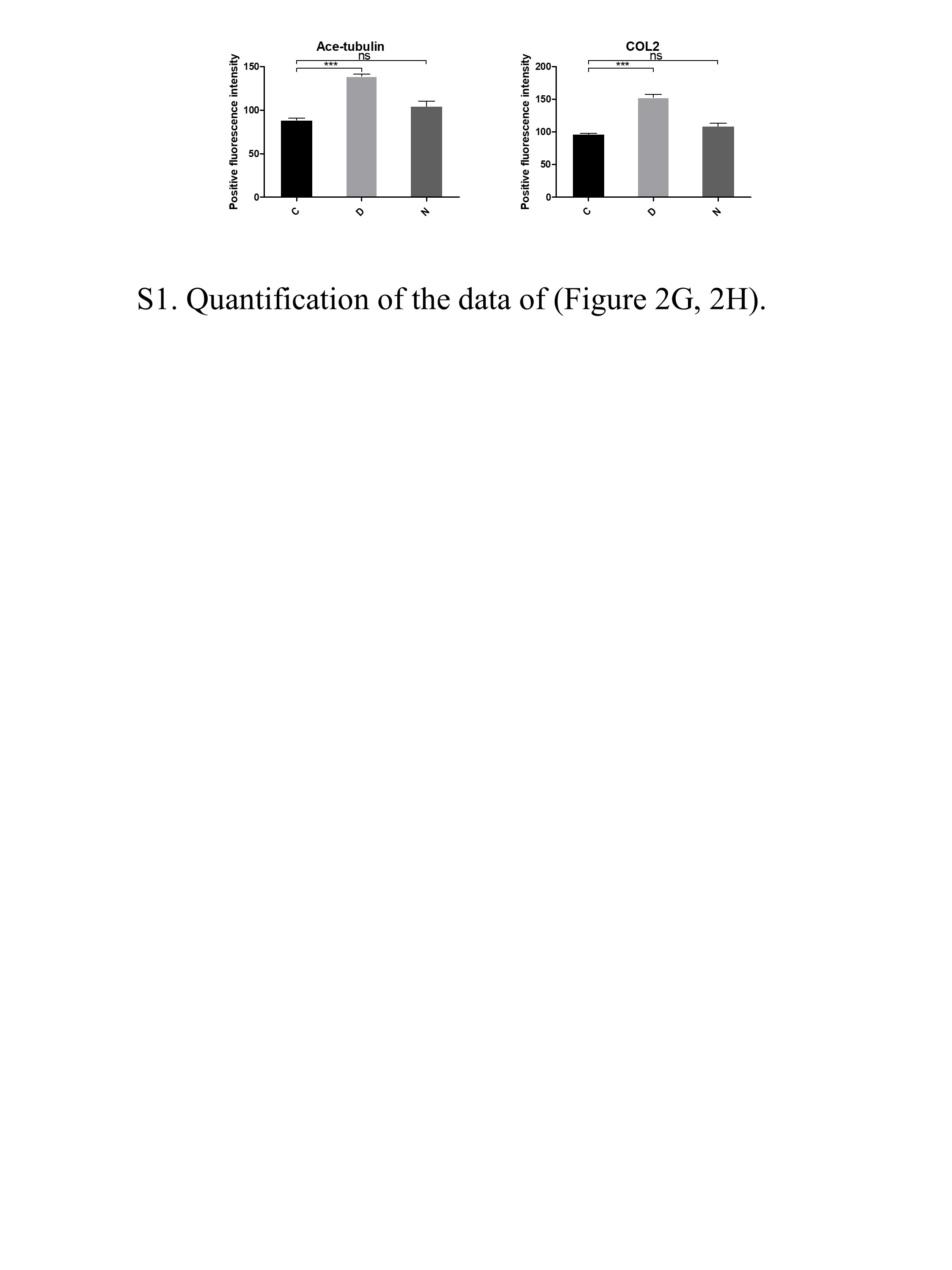

Supplement: Supplementary file 1 [file Image1.JPEG]
